# Measuring the relevance and impact of innovation and social forces for Transnational Value Chain’s embeddedness in a region

**DOI:** 10.1371/journal.pone.0291646

**Published:** 2023-10-26

**Authors:** Victor Cepoi, Alenka Pandiloska Jurak

**Affiliations:** 1 Faculty of Information Studies, Novo Mesto, Slovenia; 2 Rudolfovo—Science and Technology Centre Novo Mesto, Slovenia and Faculty of Information Studies, Novo Mesto, Slovenia; West Pomeranian University of Technology, POLAND

## Abstract

This paper focuses on understanding and explaining TVC embeddedness with the theory of Regional Innovation Systems, Social Fields, and Qualitative Comparative Analysis, which allows us to show the sufficient and necessary conditions for TVC embeddedness in a region. A qualitative-comparative empirical study was conducted in 17 regions in the form of semi-structured, expert Focus Group Interviews. The participants were regional stakeholders, representatives of supportive regional institutions, businesses, and academia, accustomed to the regional innovation processes. The findings show that the strongest effect comes from the presence of the Support for Regional Innovativeness. The presence of Innovations and networks also have to be considered, meanwhile, when it comes to institutions, the results point out that the presence and absence of Institutional framework contribute to the Regional TVC Embeddedness. Lastly, we can also highlight the absence of Cognitive Frames, which are important for Regional TVC Embeddedness. The data presented in the paper does not separate the TIER levels. Different levels could influence the conditions for the embeddedness. Half-products that are closer to the end product tend to have higher added value and are more innovative. Based on a newly developed theory and model, which focuses on social field theory and innovation, the present paper aims to test them in real-life settings. This model explains how the social forces and innovation processes influence the company embeddedness by emphasising the necessary and sufficient conditions to have TVC embeddedness.

## Introduction

Some authors consider that regional development is a process “from below”, which brings about an improvement in living conditions within the territory [1]. which is, however, also influenced by different factors in different degrees, such as cultural dimensions, demography, political circumstances and the global economy as well.

In recent years, the importance of regions has increased in various aspects in the context of economic development. Regions have attracted the attention of scholars, starting with the geographical, political, or economic and finishing with the social importance that regions bring to the development of society. Thus, the importance of the mezzo-level study of various processes is at the forefront. The social complexity of daily interactions (political, economic, and social) requires us to find suitable approaches to explaining these processes to gain the most out of them. As a result, the nation-states have partially started to lose their influence and legitimacy [2], giving the lead for spatial formation through the framework of economic and cultural transformations.

Following this line of argument, the regional level becomes the platform for creating innovation with the help of the socio-economic context. The regional socio-economic context can be regarded as the engine for development in a given milieu [3]; as the authors point out, the regional level is important for the innovation system due to the intra- and inter-regional knowledge flows. When scholars talk about local and regional economic trajectories, regional connectivity is at the forefront. Thus, scholars understand that the spatial extent, nature, and directionality of the flows connecting each region constitute an important piece of the global economy [4]; as the authors point out, regional development is keen on connectivity, which is also a way of diagnosing the shortcomings and opportunities in a given region. To do so, there should be a consideration of the regional intensity/magnitude, its spatial extent, directionality, and nature in terms of business functions. As a result, a region’s possibility of implementing and governing systematic integration (including coordinating a diverse structure of localised and non-spatial value networks) determines global connectivity.

At the same time, it became very important, how new knowledge and technologies get into the region to enable regional innovation. Transnational Value Chains and companies, that are members of these chains take an important part in this. There is not only a pool but also a development of new tacit knowledge and technology, circulating internally in the respected companies (see [5]). The issue is, how, when and if there is also a knowledge spillover and technology transfer happening in a relationship between companies from top to bottom. Namely, does a company that is at the end of the value chain and manages the cain only buys half/products from suppliers (e. g. Tier 1, Tier 2, Tier 3…) or is it also involved in their R&D. That insinuates, in what extend the TVC is embedded in the region.

TVC embeddedness can be considered a complex phenomenon that requires a detailed analysis of all its related factors. For example, we might emphasise that the TIER level of a given region, which implies the power relationship of the region in a given TVC, is an additional asset to be considered when discussing TVC embeddedness in a region. Bearing in mind the above-mentioned, in this article, we focus on understanding and explaining TVC embeddedness in a given region from a different angle. To do so, a new explanatory model with a particular focus on social fields and innovation theory was developed during the Jean Monnet Center of Excellence, Technology and Innovations in Regional Development for Europe 2020 (TIR2020) [6] project. The focus is on conditions that influence specific regions for TVCs to become more embedded in respected regions. Thus, we consider the theory of Social Fields, which enables us to explain the interplay between actors and social environments as social arenas or local orders [7] to explain the TVC embeddedness in a given social milieu. As Beckert [8] asserts, the market of fields, which consists of three social fields (institutions, social networks, and cognitive frames), has the power to explain an economic outcome.

Nevertheless, Beckert also points out that this approach considers the social forces’ interrelations as sources of field dynamics. With reciprocal influences in the market fields, Social Field Theory and the social forces have been shown to explain very diverse economic phenomena (in our case, the TVC embeddedness) but also the relationship between SMEs and high-performance computing (HPC) academia [9]. To have an explanation of the phenomenon from a different angle and test the proposed model, our starting point is derived from our research question:

*What are the prerequisites for TVC embeddedness in the region?*

As a result, our research objective is to adapt the Social Field Theory and explain the TVC embeddedness in different regions. To do so, in the following chapters, we will focus on the theory of TVC, Social Fields and Innovation. With the help of Qualitative Comparative Analysis (QCA), we will show the sufficient and necessary conditions for TVC embeddedness in a region.

Considering the novelty of the tested theory and model, we opted for an extensive literature review. Thus, it allows us not only to have a better understanding of the new model but also to understand the selection of the methodology to test the model.

## Theoretical framework

### TVC and its regional embeddedness

The simple term ‘embeddedness’ holds a very complex set of concepts. After exploring the explanation of the theorem, itself and reading relevant scientific articles, it remains an ambiguous concept. It is used in different scientific disciplines. The concepts found in the literature are mainly diverse according to the type of network in which one operates, as well as with whom and at which level one has relations (for a more extended view, see [10]). This article does not address any new aspects of the explanation of the theorem. We acknowledge the scientific findings and choose to focus on Polanyi’s meaning. As Beckert [11] explains, Polanyi understands embeddedness in a twofold manner. He sees markets as necessarily limited by institutional regulations that connect them to the moral fabric of society and views the reference point of embeddedness as not the economy as such, but ‘the larger social systems in which all economies are located’ [12]. We also consider Granovetter’s observation that all economic exchanges are necessarily embedded in social networks [13]. Polany’s idea on double movement is very important when considering shaping and influencing the market, moreover how and if the other actors as regional, and national authorities regulate the market. However, in this paper, we do not deal with the field of market regulation but with knowledge spill-over and technology transfer, which happened among companies and regions.

Furthermore, when addressing embeddedness concepts, in this article we are focused on the vertical and horizontal dimensions of embeddedness. Vertical embeddedness is seen as relations between different identifiable levels in a network and can be distinguished geographically, based on channel structure, or within a specific business. The horizontal dimension is seen as the relations of the actors within a specific network level. Business actors may be embedded in various competitive or cooperative arrangements, such as strategic partnerships, joint ventures, or similar [10]. In this article, we are interested in the different layers of the relationships a business or one firm can have, so we take the complexity of the network dynamic into account.

In our perspective, the multivalent embedding principles (social, economic, political, and cultural) point to at least some reliance, but most critically interdependence, between the linked structures or units. In other words, at least one of the embedded actors should consider how the other’s actions have an impact on their own when making decisions. We do not equate the phrases embedded and dependent in this context; rather, we only stress the need of realizing that embedding is a process with repercussions. The weight of the circumstantial relationship is imposed on the players through the development of a stronger tie. It implies a logical need to revisit the exogenous variables of regional background in the context of regional development (e. g. [14–16]).

### Determinants of TVC embeddedness

In Gertler, Wolfe, and Garkut [17], one can read that the research shows that the national economy and national systems of innovation consist of a series of regional and local systems of innovation integrated directly into the global economy. Regional and local economies display many characteristics relevant to sustaining innovative practices in a global economy (ibid). Furthermore, innovation has been more accurately shown to be (in many cases) non-linear, iterative and interactive–that is, a social process that is often triggered by consumers (or ‘users’) who engage in a mutually beneficial dialogue and interaction with producers but can also be determined by relationships between industry and academia [18] or relationships between triple helix actors [19]. In this way, users and producers actively seek to learn from one another in a process called ‘learning-through-interacting’ [20, 21]. Deriving from that, we can agree with Gertler, Wolfe, and Garkut, in their assertion that the importance of the region arises from the emphasis on the nature of learning in the global economy [17].

More and more, multinational corporations are exploiting technology globally and gaining access to new technology around the world through the diffusion of R&D and increased collaboration. In the case of dominant companies, technology cooperation is a tool used to have access to and use the technology capabilities of its suppliers [22]. Companies competing on a global basis are establishing their research activities in key R&D centres distributed across several countries and are building alliances with university research centres, as well as with other firms offering complementary knowledge and skills [23].

Considering the supplier-firm mutual benefit in raising competitive advantage and beneficial technology and knowledge transfer between firm, supplier, and region, practices that do not take advantage of that still seem to be more as a rule and less as an exception. The results of the study, made by Gertler, Wolfe, and Garkut [17], indicate that the home base is still the primary site for innovation. It is suggested that the home region remains the principal site at which firms engage in local learning-through-interacting; in contrast, foreign-owned firms, by and large, do this at home. As Gertler, Wolfe, and Garkut conclude, ownership continues to matter, and we do not seem to have achieved a post-national system of innovation [17].

**Fig 1:**
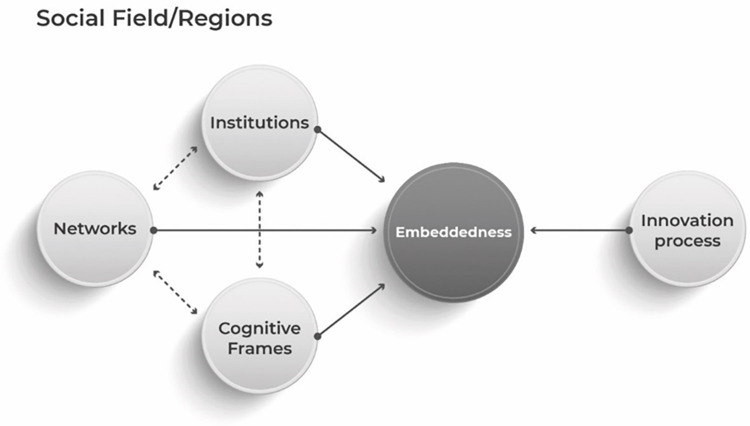
Regional TVC embeddedness model. Source: Authors’ visualisation.

To measure whether there are relevant social forces that influence the TVC embeddedness at different levels, we propose a model (see Fig 1), which includes social fields (can be understood in both broader and specific territorial space, e.g. regions, which suit the purpose of this research), innovation process and social forces (institutions, cognitive frames, and networks).

### Regional innovation process

Regions start to grasp greater importance when it comes to the development of the political, social and economic milieus. The shift to the regional perspective offers the possibility to create a favourable socio-economic framework for innovation development. As Dodescu and Chirila [3] highlight, this socioeconomic framework is considered one of the engines of development in a given space. Furthermore, the focus should not rely only on the aspects that are happening in a localised given space but rather consider the intra- and inter-regional knowledge flows. As a result, this underlines the importance of a region within the Innovation Systems. Nevertheless, this process should be done in isolation from broader social goals. As a process/ interaction between different stakeholders, the relationship between the regional level development and the innovation process should be determined by concrete mechanisms [24].

### Regions as social fields and its forces

Development is bound to the process of innovation. It is considered a vital aspect, especially for companies, because of the increased role of knowledge in the present information era. Thus, with the help of this knowledge, companies can attract and retain their competitive advantage [25]. As a result, it determines the need to be innovative constantly. The complexity of present political, economic and social relations determines our daily and industrial interactions. This argument is also valid for explaining the technological transformations, which require the interactions between different stakeholders from the political, economic, and social environments. As a result, with the constant technological progress, it is of utmost importance to understand it. Considering the importance of technological progress and its effects on regional development, mainstream economic approaches can no longer explain all the changes that are happening. Thus, scholars must adopt a broader explanation model for innovation processes, which are considered the promoters of economic, technological, and regional development around the world to keep up with the changes. It is considered that the efficiency of the national research, the high added-value technologies, the transfer mechanisms available in a given region, and the presence of innovation eco-systems are correlated with research and innovation performance [26]. As the authors mention, the low-performing European Member States and regions need support for the improvement of their research and innovation systems and policies, including their infrastructure.

Considering the literature related to innovation processes, either deals with case studies (community or regional level) or does not always consider all the aspects that trigger innovation [27, 28]. As a result, it is of utmost importance to grasp other explanation models, especially for the cases in which the basic economic models do not function. In general, innovation processes operate within a specific market, which determines the operational aspect of any innovation process. Any given market is both politically and socially constructed. As Fligstein [29] argues, social construction reflects, firstly, a country’s culture and its history of class relations and, secondly, the government’s interventions. As such, at the moment the capitalist national system is understood as a whole, these processes are linked to culture, capital allocations, labour relations, tax systems, and state involvement and ownership in key industries. To have a better understanding of the complex interplay of mechanisms that determine the political and social construction of any market, it is necessary to link it to a theoretical framework. Thus, the theory of Strategic Social Fields considers all these aspects and acknowledges the interactions on different levels (e.g., national, regional, etc.) [7]. As Fligstein and McAdam point out (ibid.), the theory focuses on the social arenas or local orders, which are seen as the environment for interaction between actors and social environments. Within these fields, different actors interact under a set of common understanding of the relationships, purposes, and rules of the fields. States, regions, or any environment are considered a complex system of fields, regarded as local orders. Thus, we do not have to consider the boundaries of Social Fields as static or fixed, but rather shift depending on the definition of the situation.

Moreover, if we consider any market as a field or social order, we have to consider that within the arenas, actors gather and frame their actions vis-à-vis one another [29]. The interactions are determined and explained with the help of social structures on individual actions. More specifically, the theory considers three social forces relevant to economic outcomes: institutions, social networks, and cognitive frames [8].

As Beckert points out, the three social forces are not autonomous; they are in a relationship of reciprocal influence. Institutions influence the structure of social networks; simultaneously, for cognitive frames, it makes certain cultural meanings socially relevant. From the perspective of social networks, on the one hand, these establish power to shape institutions and, on the other, shape and diffuse cognitive frames. Lastly, cognitive frames also shape perceptions of network structure and provide legitimation and shape perception of institutions.

#### Towards TVC embeddedness through regional formal institutions

As mentioned earlier, one of the forces that influence regional development and the innovation processes in it are the formal institutions. These can be understood as: ‘[…] a set of rules, compliance procedures, and moral and ethical behavioural norms designed to constrain the behaviour of individuals in the interests of maximising the wealth or utility of principals’ [30]. In other words, as Jakobsen and Aarset [31] point out, it refers to the political-administrative regulations, which are provisions and laws. Thus, these provisions and laws influence the behaviour of the economic actors. The influence can have a positive impact through the protection of the costly and high-risk innovative processes, especially in the first period of their emergence. Additionally, some research and development (R&D) activities must be protected; thus, this initiative stimulates companies to make investments and promote innovation processes (ibid.). In contrast, the regulatory framework of a given country or region can also hinder the process of innovation. As the authors argue, these regulations can be of restrictive origin. For example, the market can be burdened by limiting fair competition. As a result, it impacts the strategies that any given enterprise has. Nevertheless, at the moment these obstacles are removed, specifically, by allowing and providing the necessary regulatory framework for fair competition, innovations and all processes related to them will thrive (ibid.).When it comes to the innovation processes, there can be mentioned the role of institutions in creating a favourable environment and opportunities for innovation [32]. Additionally, according to the report, institutions contribute to the development of innovation with the help of formulating innovation policy or conducting basic and applied research or helping technology diffusion and encouraging technology entrepreneurship. As Huggins and Kitagawa [33] point that regional innovation policies and regional stakeholders in science and technology are the pillars for the creation of the appropriate context for knowledge transfer. There is a difference between western and transitional economies when the focus is on institutional quality. For example, for western economies, at the forefront are incoming foreign direct investments and technologies, meanwhile, transnational economies focus on productivity and spill-overs [34]. Even more, western economies are characterized by having high levels of institutional quality, namely institutions characterized by an effective regulatory framework. As a result, we can mention that economic processes are composed of processes of coordination between rules. This is needed at the moment agents do adopt the rules in the same manner. Additionally, the de-coordination and re-coordination of the innovation system originated, pinpointing the diversity of rules in the innovation system that compete with each other [35].

#### Social networks

Another social force that has to be considered in explaining any economic interactions are social networks. It influences the process of development in various ways. For example, networks aim to increase the interaction between different stakeholders, even if there is a concern about using technologies. At present, different stakeholders are connected, small and medium enterprises (SMEs) generate knowledge and profit-sharing from complementary competencies [36]. The link between stakeholders offers SMEs the possibility of increasing their importance in the market, because of these innovative new products and processes, but also these links are considered the engine of technological progress and economic growth [37]. On the other hand, regulatory intermediaries can have a positive or a negative role as a bridge between value chain leaders and material providers [38]. Nevertheless, it is important to mention in this context that the size of the company is important for performance, behaviour and networking [39].

At present, with the increasing influence of the globalisation processes and the fewer barriers for knowledge and information diffusion, in the literature, there is a new paradigm for innovation–open innovation. By open innovation, one can understand: ‘the use of purposive inflows and outflows of knowledge to accelerate internal innovation and expand the markets for external use of innovation’ [40]. At the emergence of the new paradigm, open innovation was understood as a collaboration between two enterprises, which opened the internal process of innovation, as opposed to the closed innovation model, in which internally developed products and services remain in the enterprise [41]. To gain popularity among scholars, the new paradigm of innovation focuses on three main pillars:

1. understanding innovative ideas as coming from the outside of the enterprise,
2. the framework of open innovation builds on the idea of emphasising the importance of challenges that firms face in capturing returns from their innovative effort,
3. the role of the business model is to mediate between the technical inputs and economic benefits of technology by structuring how a firm created and captured value from a specific market [42]

Additionally, it orchestrates the role of different stakeholders in the innovation process [41].

Networks have the aim to boost the interaction between various stakeholders, even if there is a growing concern about the usage of technologies. With the help of networks, one of those that benefit from the interconnectedness is the SMEs. At the moment stakeholders link with each other, the SMEs generate knowledge and profit [36], making them engines of technological progress and economic growth [37].

#### Cognitive frames

Finally, the last social force that Beckert talks about is cognitive frames. The impact that globalisation has on knowledge and its sources and innovation expansion was mentioned earlier. Within the literature, the highlight is on how globalisation and national culture exert influence on R&D performance. For example, any company should consider the national/ regional milieu at the moment they want to innovate. Thus, the milieu should be favourable to innovation because, in some cases, at the moment a national/ regional culture is undermined, innovations can be rejected regardless of whether the number of resources spent on innovations is high [43]. There is still no common and precise definition of culture, thus making it difficult to examine it concerning innovation [44]. Nevertheless, the role of culture is to diffuse, activate, and select from among available representations when it is helped by institutions, networks, and social movements [45]. Thus, scholars highlight the need to understand and develop the idea of culture, as an interaction of shared cognitive structure and supra-individual cultural phenomena, in the form of media messages, conversations or material culture that activate the cognitive structures (ibid.).

Considering all the above mentioned, we can argue that the interplay between institutions, networks and cognitive frames form a given field, where the innovation processes occur. As mentioned earlier, this theoretical understanding can set a new approach toward understanding new economic realities and all development processes that occur in a given field. This theory allows us to provide comprehensive in-depth knowledge about all relevant aspects of the innovation and HPC landscape in each of the three pilot regions.

It is considered that besides the presumption that only economic aspects are important for the economic processes, the cultural background and the values society has, are also part of these processes [46]. Even more, in the literature, the focus on cognition as a factor in explaining economic results is of a less significant role [29]. At the same time, the change in cognitive frameworks leads to a change in the perception that networks and institutions [8]. Nevertheless, it is important to mention that studies show that the cultural aspect of a given society influences R&D performance. An eloquent example is the company’s desire to innovate. Before proceeding with the innovation process, it must consider the national aspect of the environment and see if it is favourable for innovation [43]. At the moment the company does not consider the national aspect, it might lead to the rejection of the innovation process, even if the company’s investment in innovation is high [44].

## Data and methodology

The data were collected within the framework of the project Jean Monnet Center of Excellence, Technology and Innovations in Regional Development for Europe 2020 (TIR2020) [6]. The research uses third-party data. The data comprises questions that assess the level of Innovation. Institutions, Networks, Cognitive Frames and TV for 20 regions from 20 different countries in Europe (each indicator is measured through a set of 4 questions with labels). For each question, the participants agree on a single label (score) from 1 to 4, which is used for further analysis. In order to obtain the data, interested parties need to contact prof. Roncevic (borut.roncevic@fis.unm.si), the holder of the Jean Monnet TIR 2020 project, as the data availability is bound to the project contract with European Education and Culture Executive Agency. Also, to get access to the repository, interested parties can register through the following link: https://www.tir2020.net/recent-analysis-results.

During the project implementation, we performed semi-structured, expert Focus Group Interviews within multiple European regions, rating from poor to developed regions on the continent. The regions selected for this research are: Cluj County, Romania; Nord Region, Republic of Moldova, Zakarpatya, Ukraine; Lazio County, Italy; Braganta Region, Portugal; Vienna, Austria; Northern Savonia, Finland; Overijssel, Netherlands; Klaipeda, Lithuania; Nord-Norge, Norway; Vidzeme, Latvia; Vojvodina, Serbia; South-East, Ireland; Nordjylland, Denmark; Sofia Region, Bulgaria; Continental Croatia, Croatia; Moravian-Silesian Region, Czech Republic.

The authors of this paper did not seek the approval of the institutional committee to approve the study. The study does not fall under the WMA Declaration of Helsinki, because it is not medical research and does not include research on identifiable human material and data. The study did not involve patients. Interviewees were representatives of the business sector and supporting organizations. Nevertheless, all steps to assure the GDPR regulations were taken. The results were anonymised. The anonymized survey was archived later, and the interview notes, transcripts and recordings from interviews are also archived without any personal data. Data collection was done in a way where the informants report on a narrow field and therefore include no personal information since we are not interested in the informants as such. We are strictly limiting the information gathered to the specific field of interest (the respondent’s answers relating to their knowledge, skills and competencies in regard to business and regional development. The coding key was deleted. Without this coding key, it is impossible to recognise any identification of interviewees and not possible to reconstruct by authors of the study or anyone else. Data, used for the paper was taken from the anonymised database. Interviewees were invited to participate in the study with a letter, specifying the research, research purpose, and how data will be collected, stored and used. Their consent was given in verbal form before the interview began. It was written in transcripts, which were later deleted to comply with GDPR.

The data collection tool is heavily based on the Social Field Theory with conceptual coalesce to the Regional Innovation Systems and Transnational Value Chains Theories. The goal was to identify the relevant aspects regarding the Innovative Capabilities of a region, as well as the Importance of Regional Institutional Support, Network Contribution, Cognitive Frames and TVC Connections for the innovative potential. Participants (regional stakeholders, representatives of supportive regional institutions, businesses and academia, accustomed to the regional innovation processes) were selected after desktop research concerning the regional context, supported by using the ‘snowball’ technique, which helped identify the necessary experts for a thorough assessment of the regional contexts. Upon discussing the relevant aspects, respondents were asked to agree unanimously on a labelled score (1–4), resembling different degrees of development of the phenomena, for each question. These scores represent the quantitative data collected during the project, the same data we used in the context of this research.

The questionnaire consists of twenty questions covering different concepts of regional development and regional innovativeness, which became part of our research model. The questions form five main groups: Institutional, Networking, Cognitive, Regional Innovative, and TVC-related particularities. Each of the main concepts was associated with batteries of four questions per phenomenon:

- **Regional Innovation Capability:** *Innovation Level; Information Circulation; Enterprises’ R&D involvement; Open Innovation*
- **Institutional Support:** *Institutional support for innovation; Attraction of Talented People; Retention of Talented people; Innovation Policy Status*
- **Networks:** *Network Contribution to Innovation; Regional Actor’s cooperation; Cooperation with Outsiders; Trust*
- **Cognitive Frames:** *Entrepreneurship and Creativity; Importance of Education and learning; Competition; Globalisation*
- **Transnational Value Chains:** *TVC Support for Regional Innovativeness; Economic Relationship between Region and TVC*
- **Regional TVC Embeddedness**: *Degree of Regional Embeddedness with TVC; the role of Transnational Value Chain (TVC) relationships; Extent to which the regional economic performance depend on Transnational Value Chain (TVC) relationships*
- **Region TIER**: *Regional Role in TVC*

In the context of the present research model, we used the sub-concepts as independent conditions for analysis. The exception is the TVC Embeddedness (the outcome). To quantify the TVC Embeddedness, we merged the three sub-categories (degree of embeddedness, economic relationship, and innovative support) into an aggregate score for each region. As a grouping factor, the question assessing an average regional TIER within a TVC was used separately for further analysis.

Considering that we have 17 observed cases in our dataset, Qualitative Comparative Analysis was used. This method is suited for small-N comparative study [47]. Additionally, QCA allows the use of both the theoretical framework and empirical data. Thus, the theoretical framework determines the research problem within our research, and we continue with the specification of conditions for the desired outcome [48].

Furthermore, QCA allows us to operate with the membership or non-membership status attributed to each case. At this stage, the ‘raw data’ is calibrated. Schneider and Wagemann [49] mention that with the help of the calibration process, each case will receive a degree of membership in a given set. The membership score can have a value between 0 (full non-membership) and 1 (full membership). For example, a value of 0.15 indicates that the sets are more ‘out’ than ‘in’ in the membership, while a value of 0.75 would indicate that these are more ‘in’ than ‘out’ [50].

One of the aims of the QCA is to address the absence of certain conditions to have a presence of something. E.g., to have democracy, there shouldn’t be any corruption in a country. Thus, it shows the importance of not having corruption to have democracy. Otherwise, if there was corruption, there would not be a democracy. In line of the argument, within this paper we demonstrate that it is important not have certain conditions to have embeddedness. This result also confirms the accuracy of a newly developed/proposed model.

This method allows us to determine sufficient or necessary conditions for any particular outcome [51]. Namely, for our model, we will be able to determine if regional innovation, institutions, networks and cognitive frames determine the regional TVC embeddedness as an outcome. At the same time, it will rely on parameters of fit: consistency and coverage. Consistency indicates how closely a perfect subset relation is approximated with a given outcome, while coverage determines the empirical relevance or importance of an outcome by a condition or combination of conditions (Ragin, 2008, p. 44). In the literature, there is no sole answer for setting the threshold for consistency and coverage. As Schneider and Wagemann [52] emphasise, the thresholds vary depending on the research design, such as the number and knowledge of cases, quality of data, and similar factors. As a result, for a condition to be considered either sufficient or necessary, the threshold for consistency is set at 0.75, meanwhile for coverage 0.5. These anchors can be considered dynamic, thus securing the casualty from the perspective of the closeness of approximation. In contrast, they also enable us to secure the empirical relevance of the conditions. As Schneider and Wagemann [49] highlight, priority has the consistency parameter, because it makes sense to consider the coverage of a condition as being consistently sufficient or necessary.

## Results: TVC embeddedness and its conditions

As mentioned above, in our proposed model, TVC embeddedness is at the forefront; meanwhile, Innovation, Social Forces (institutions, social networks and cognitive frames), TVC Regional Innovativeness and Economic Relationship between Region and TVC and Regional Role in the TVC are considered as conditions to have Regional TVC Embeddedness. Thus, the analysis has pointed out several aspects that must be considered when we want to opt for a TVC embeddedness in a given region (see Table 1).

**Table 1:** Sufficient and necessary conditions for TVC embeddedness.

	CONS.SUF	COV.SUF	CONS.NEC	COV.NEC
**Innovation**	**0.812**	**0.515**	0.515	0.812
**Institutions**	**0.859**	**0.505**	0.505	0.859
**Networks**	**0.831**	**0.693**	0.693	0.831
**Cognitive Frames**	0.716	0.555	0.555	0.716
**Transnational Values Chains**	**0.965**	**0.868**	**0.868**	**0.965**
**Region Tier**	**0.806**	**0.609**	0.609	0.806
**~Innovation**	0.583	0.837	**0.837**	**0.583**
**~Institutions**	0.529	0.784	**0.784**	**0.529**
**~Networks**	0.487	0.602	0.602	0.487
**~ Cognitive Frames**	0.581	0.753	**0.753**	**0.581**
**~ Transnational Values Chains**	0.476	0.557	0.557	0.476
**~Region Tier**	0.521	0.686	0.686	0.521

Source: Authors’ calculations

The results point to several aspects that have to be emphasized. Firstly, the strongest effect comes from the presence of Support for Regional Innovativeness. These results show for the selected cases that at the moment a certain milieu receives support (either it Is an institutional support or support from other parties) for improving the innovation conditions, these will be a positive trigger for the Transnational Value Chain embeddedness. It creates favourable conditions for the regions to become more competitive and could allow changing their TIER level in time. Meanwhile, when it comes to Institutions (as a separate condition), the results point out that the presence and absence of an institutional framework contribute to the Regional TVC Embeddedness. Thus, when we have the combination of support for innovation, attracting talented people, and the presence of the innovation policy, it creates the embeddedness effect, whether these are present or not in the region.

Secondly, the presence of Innovations and networks also has to be considered. If in the previous paragraph, the focus was on the support for innovations, here the focus itself is on innovations as an occurred outcome. On one hand, we talk about innovations being the driving force for the embeddedness of TVCs in different regions. On the other hand, the presence of networks also contributes to embeddedness. The rationale behind this result lies in the fact that the more connected the stakeholders in a specific region with foreign actors, the greater development of a TVC is possible. Even more, it can increase the embeddedness of the TVC in the region. Thus, policymakers should also focus on aspects such as the cooperation of stakeholders in a region, cooperation between regional stakeholders and outside the region, and not lastly it is important to make improvements toward creating a trustful environment between the above-mentioned actors.

Lastly, we can also highlight the absence of Cognitive Frames, which are important for the presence of Regional TVC Embeddedness. In other words, considering the conditions that formed Cognitive Frames—creativity and entrepreneurship, regional attitudes/culture enable learning processes in the region, competition and globalization, seem not to play a vital role for TVC embeddedness in a given region.

If at first glance, a part of the above-mentioned story does not make any sense or even comes into a contradiction with the obtained results, a closer look highlights some exciting aspects to achieve TVC embeddedness. The presence of TVC in the region is both sufficient and necessary for the embeddedness of our case studies. The presence of an innovative region and two social forces (institutions and networks) are sufficient conditions for the region to have an embedded TVC. At the same time, a region doesn’t need to be innovative, nor have the institutional framework and cognitive frames so that TVC embeddedness is present.

Our understanding of these results can be transposed in a simple logical statement: if the TCV wants to become embedded in the region, there is no need for Innovation, Institutions, or Cognitive Frames to be present. For a region to become embedded in the TVC, it does not need grand gestures. It needs TVCs in the regions. When they have that, the region has *carte blanche* for the TVCs to be embedded. Nevertheless, it can encourage the embeddedness with innovation, institutions, and networks present.

Regions start from scratch, without innovation and institutional support. However, in time, both innovation and institutions appear and help to boost the TVC embeddedness. There is no need to have cognitive frames (competition, knowledge, creativity, entrepreneurship, globalisation) for the embeddedness. TVC can be embedded not only as business-related but also with the social component—giving money for schools, sports, and culture. Therefore, it is logical that TVCs would be more embedded or wish to be embedded in the regions that are not developed. Even more, what we can emphasise is that companies can be embedded through other different means.

**Fig 2:**
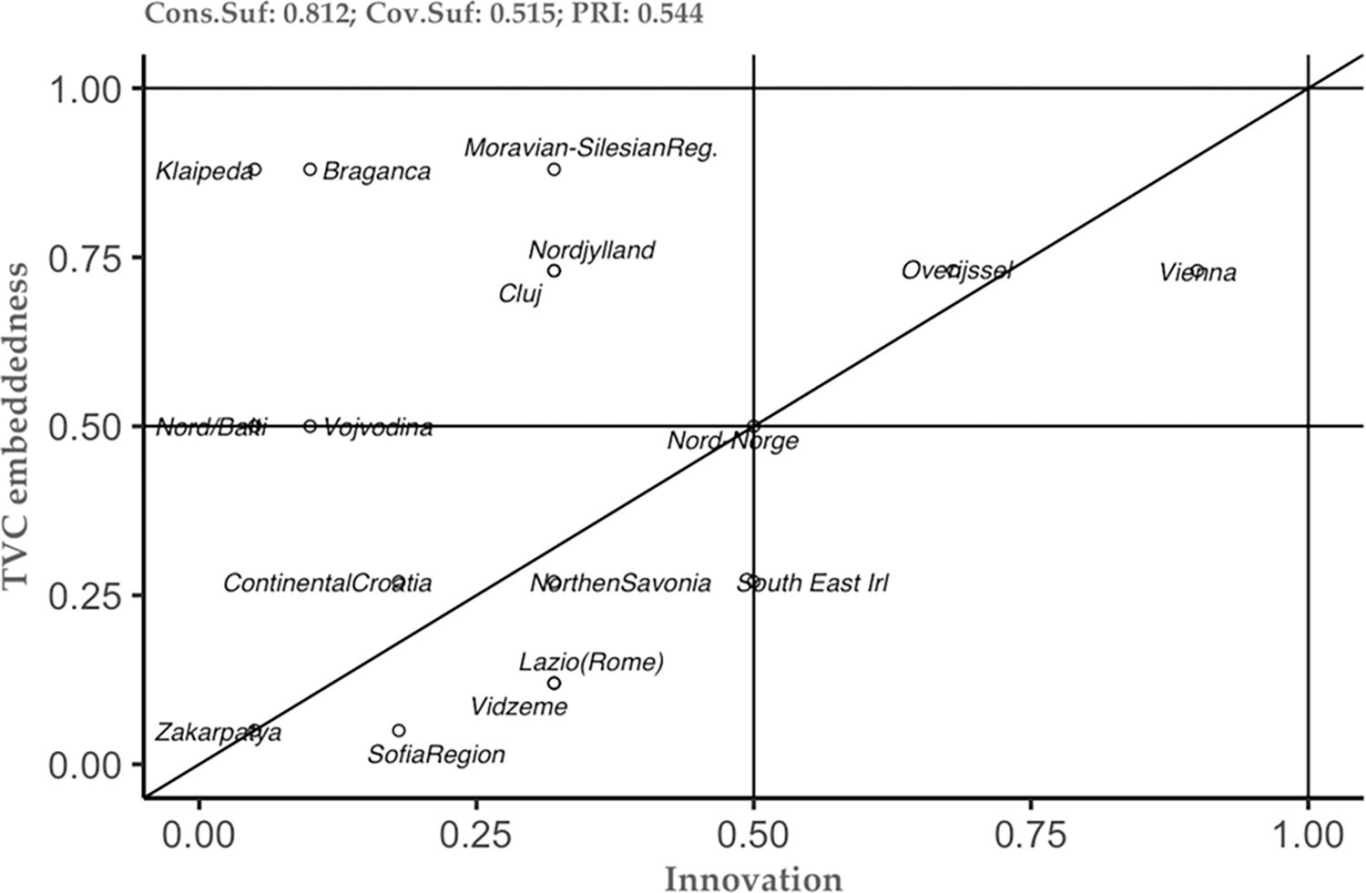
Innovation as sufficient condition for TVC embeddedness. Source: Authors’ own calculations.

**Fig 3:**
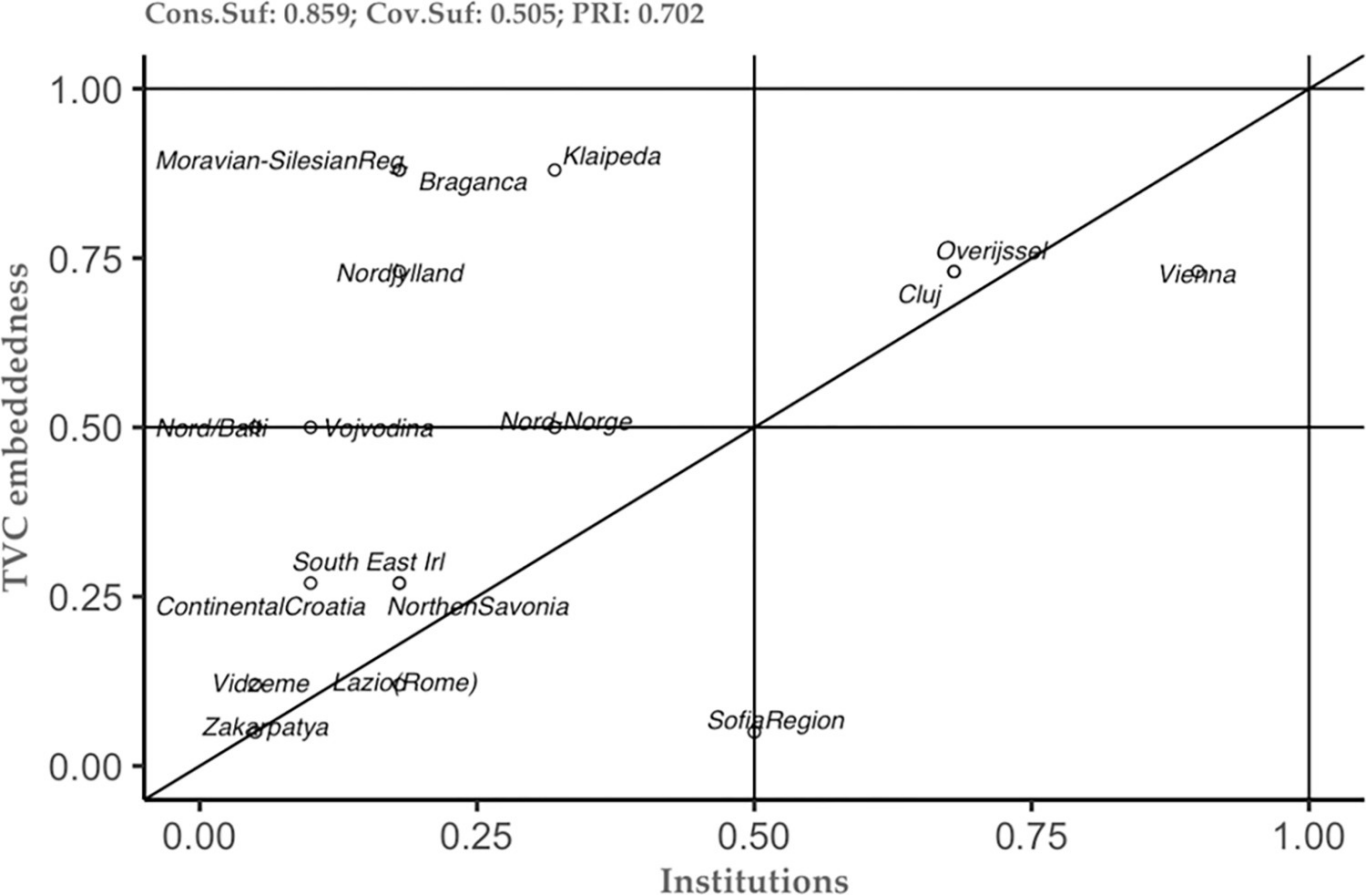
Institutions as sufficient condition for TVC embeddedness. Source: Authors’ own calculations.

**Fig 4:**
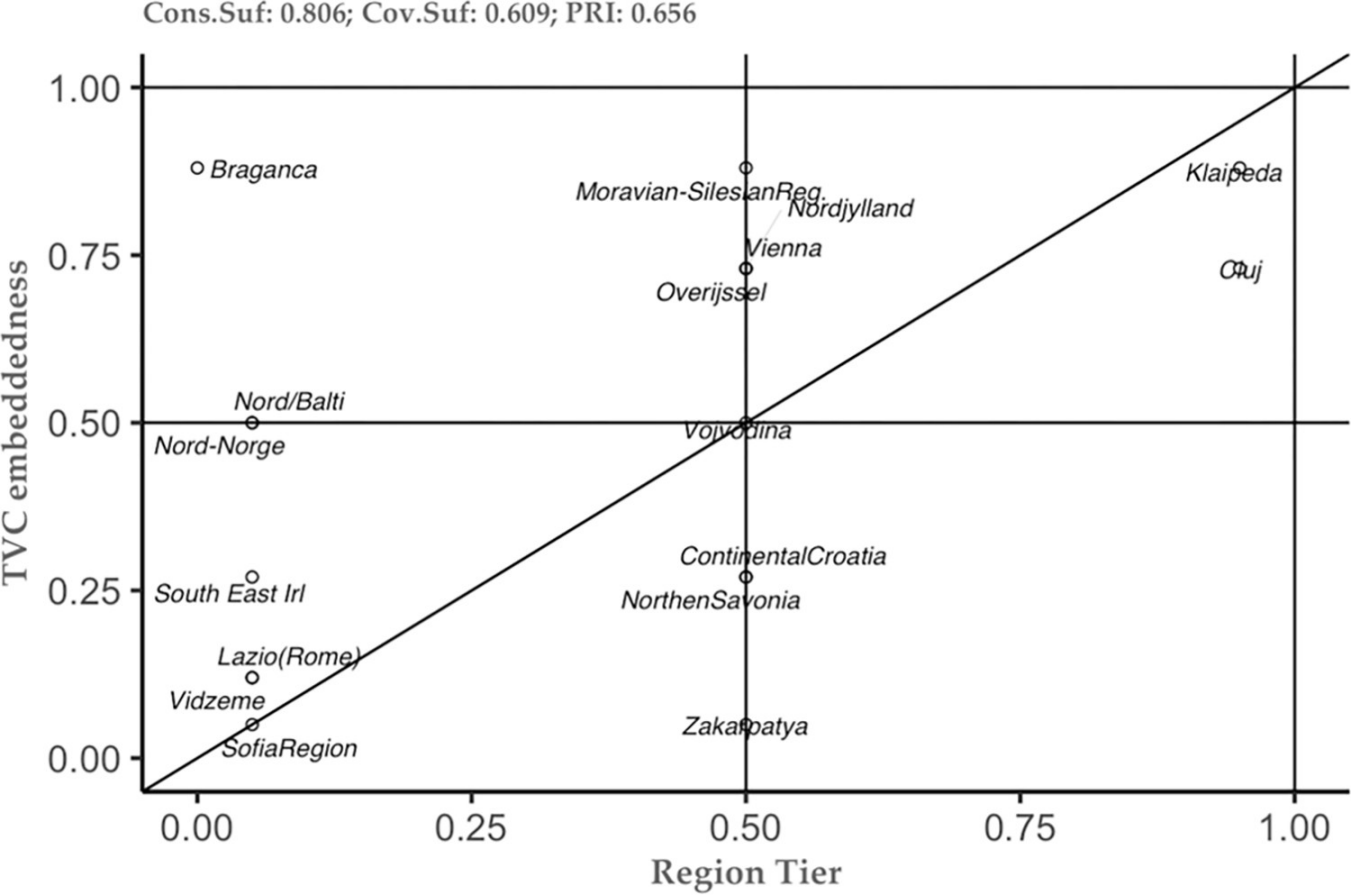
Region Tier as sufficient condition for TVC embeddedness. Source: Authors’ own calculations.

**Fig 5:**
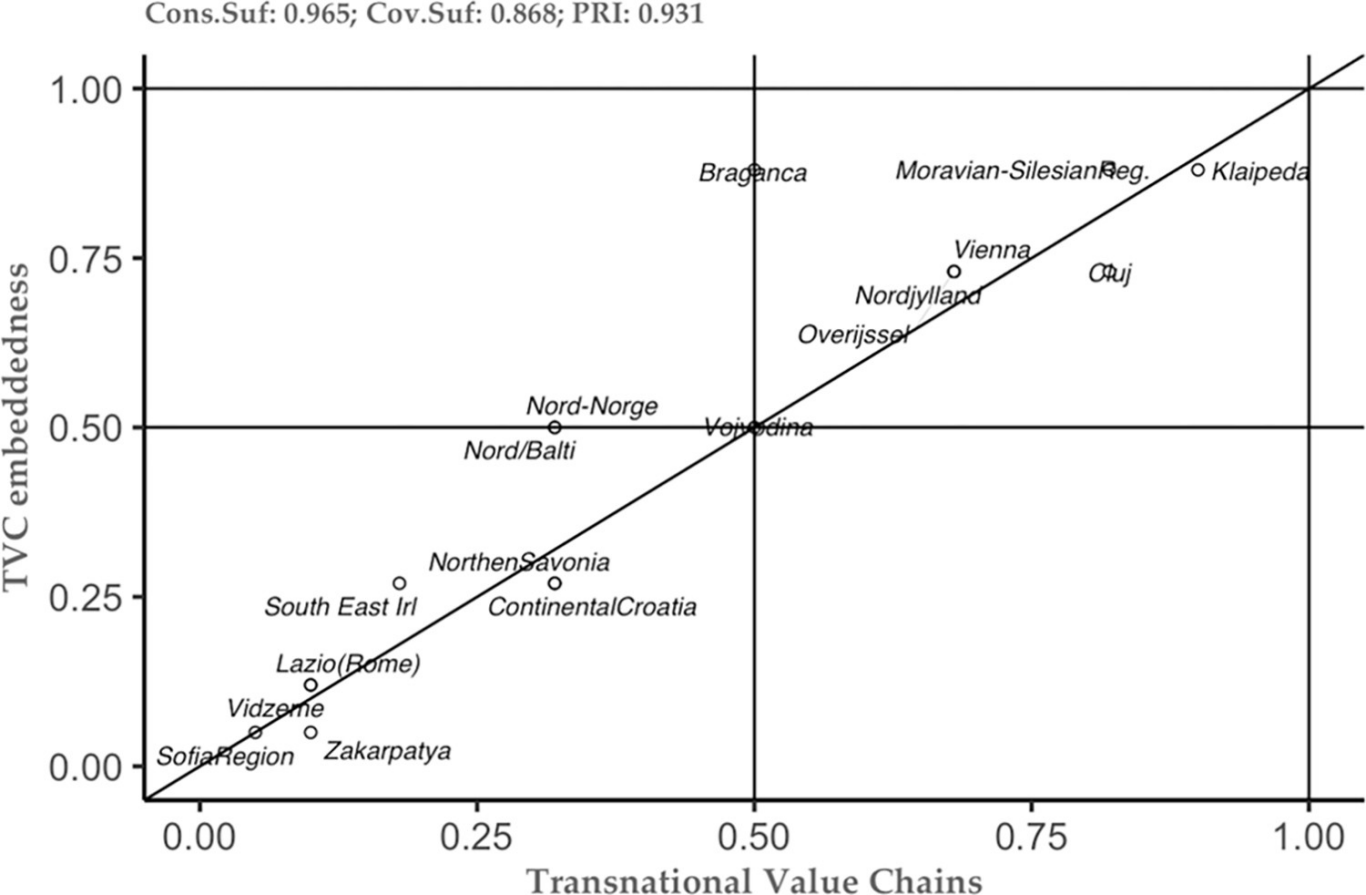
TVC as sufficient condition for TVC embeddedness. Source: Authors’ own calculations.

**Fig 6:**
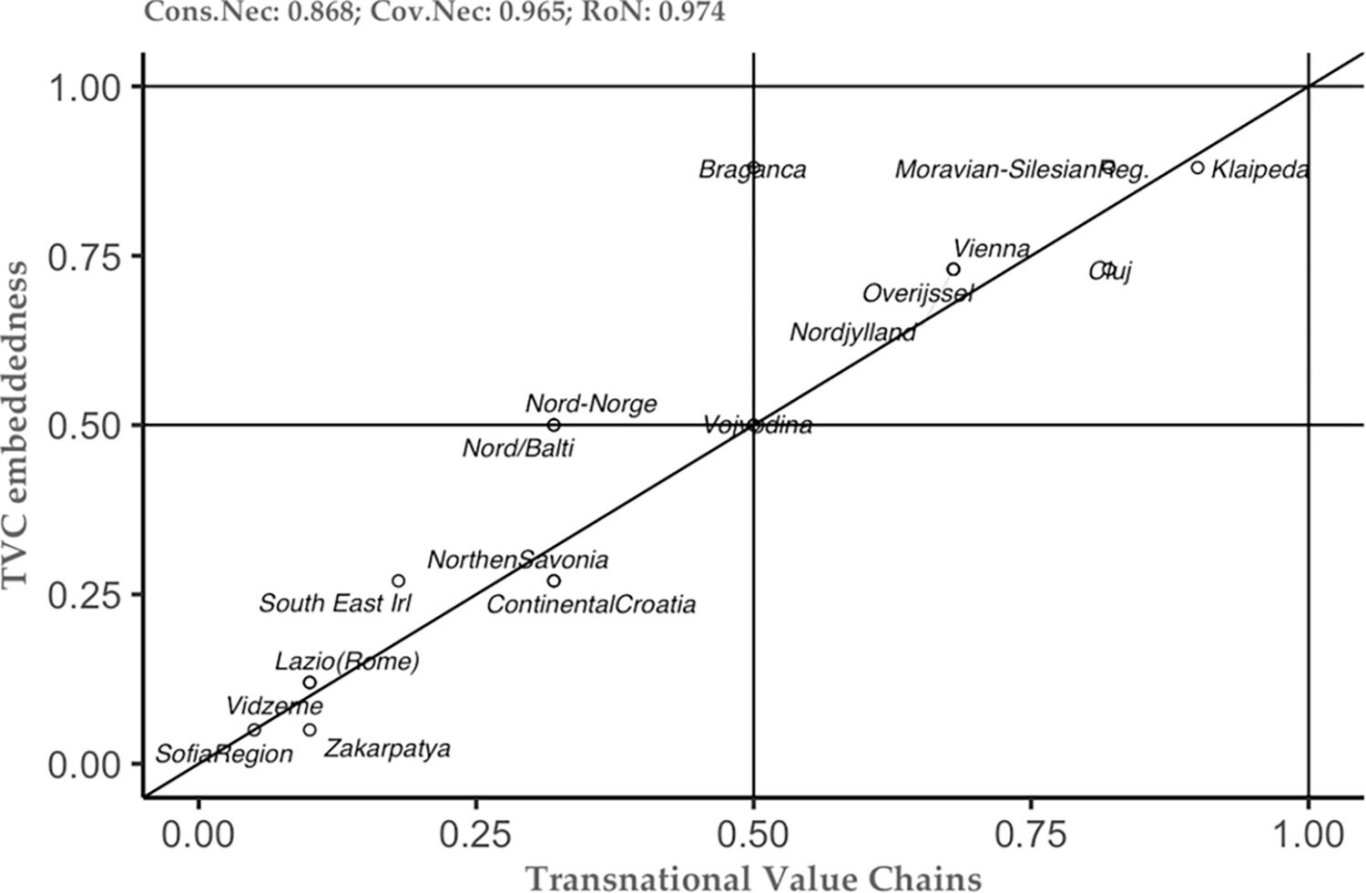
TVC as necessary condition for TVC embeddedness. Source: Authors’ own calculations.

The above-mentioned results represent just the tip of the iceberg in understanding the studied phenomenon. Thus, the combination of the new theoretical framework and the methodological approach, allows us to see not only the tip of the iceberg, but also to embrace the additional information that can help each case study separately (see [49]). To do so, we constructed several plots of the conditions that the analysis has highlighted as important (see Figs 2–6). These plots can be interpreted as follows: the plot is dived into 4 equal squares, where the cases are situated. The cases differ according to their position. Thus, if a particular case is situated in the top left square, then it is considered as a deviant case for coverage. In the moment the case is situated in the bottom right, then this case is considered as a deviant case for consistency. In the moment the case is situated in bottom left square, then we can have two situations– 1) the case is above the diagonal, then it is an individually irrelevant case and 2) the case is below the diagonal, then we have an irrelevant case. Lastly, when the case is situated in the right square above the diagonal, this case is considered to be the typical case.

As a result, it allows to have an individualistic approach to each case. This on one hand, allows to select the typical case for each of the studied conditions. On the other hand, this case-to-case approach not only offers an understanding about the phenomenon, but also can constitute valuable information for the policy-makers. As such, policy-makers have the opportunity to choose the appropriate strategies in order to boost the TVC embeddedness in a given region.

## Discussion and conclusions

As Granovetter said, all economic exchanges are necessarily embedded in social networks. This article aims to understand and explain TVC embeddedness in a given region from a different angle. The results of the research show, that the authors were correct in their assumption on what are the sufficient and necessary conditions of measuring TVC embeddedness in a region and that the proposed model is a valid measuring instrument. The results of this model explain, how the social forces and innovation processes influence the company embeddedness by emphasising the necessary and sufficient conditions to have TVC embeddedness. Namely, with the proposed model we can measure if the sufficient and necessary conditions are present in a region and if the minimal criteria is met for the embeddedness to occur or does the region need to work upon setting and improving the conditions.

In line with Granovetter’s understanding [13] of embeddedness, our analysis firstly offers the following result: if there is TVC present in a given region, embeddedness occurs; if the TVC is not present, then there can be no embeddedness. Also, as Polanyi mentions, this goes hand in hand when the focus lies on market regulations [12]. Markets are necessarily limited by institutional regulations that connect them to the moral fabric of society, and the reference point of embeddedness is not the economy as such, but ‘the larger social systems in which all economies are located’ [12]. Our analysis shows that the presence of an innovative region and two social forces (institutions and networks) are sufficient conditions for the region to have an embedded TVC; however, for a region to become embedded with the TVC, it does not need grand gestures. It needs TVCs in the regions. Therefore, the issues lie in the extent of the level of embeddedness. At this point, we could address the positive and negative sides of embeddedness as seen from previous research [17] and our data showing that the TVCs are interested only in the minimum of embeddedness. We could speculate why that is the case. Being embedded in a region would presume that the firm would act as an open system, be aware of its external environment and accept its influences as given, search for positive inclinations of the external environment and act as a responsible actor. The responsibility would need to be focused not only on the buyer and supplier but also on the social aspect of the regions with which they work, by implementing Corporate Social Responsibility (environmental aspect, philanthropy, ethical and economic responsibility). Doing so is not a self-evident attitude; it just might be, due to the lack of it, there is also a shortfall in regional embeddedness.

These findings could be the basis for further research. The received data combined with the theoretical background and previous research indicates that the model presents relevant data and can be used for further research. Consequently, using the same model, we could do more in-depth research on the necessary and sufficient conditions for each TIER separately. The results might change if we consider the TIER level.

The data presented in the article does not separate the TIER levels. Different levels could influence the conditions for the embeddedness, since the higher the TIER level, the closer they are to the end product. Half of the products that are closer to the end product tend to have higher added value and are more innovative.

Regions that want to be more innovative and raise the added value see the possibility for that to occur in the TVC embeddedness that goes beyond setting up the factory, employing people from the region and paying bills. They count on the knowledge spill-over. With that, we need to acknowledge that regions are not progressing equally [53]. The reasons have different expectations. Not all have the same start possibilities in the form of existing infrastructure, budget availability, income levels, business culture, having different development policies, and similar factors (ibid.) One could say that the expectancy of knowledge spill over would be higher in regions with lower TIER levels. So, what can be done to determine the key factors of TVC embeddedness and give knowledge to regions to become more proactive in the relevant activities? We believe that future research should focus on the individual conditions that formed the aggregated conditions (innovation, etc.) to see the differences between each individual indicator (each condition separately) of the developed tool to see which influence TVC embeddedness.
